# Akute Nierenschädigung und COVID-19: pulmorenaler Crosstalk unter massiver Inflammation

**DOI:** 10.1007/s00063-022-00919-3

**Published:** 2022-04-27

**Authors:** Timo Mayerhöfer, Fabian Perschinka, Michael Joannidis

**Affiliations:** Gemeinsame Einrichtung für Internistische Intensiv- und Notfallmedizin, Department, Innere Medizin, Medizinische Universität Innsbruck, Anichstraße 35, 6020 Innsbruck, Österreich

**Keywords:** SARS-CoV‑2, Entzündung, Sepsis, Atemnotsyndrom, Mechanische Beatmung, SARS-CoV‑2, Inflammation, Sepsis, Respiratory distress syndrome, Artificial respiration

## Abstract

Eine mit der Coronaviruserkrankung 2019 (COVID-19) assoziierte Nierenschädigung ist vor allem bei Intensivpatient:innen ein häufiges Phänomen. Das Virus selbst dürfte im Sinne eines direkten Befalls der Niere nur in geringem Ausmaß eine Rolle spielen, die mit einer schweren COVID-19-Erkrankungen assoziierte pathologische Entzündungsreaktion dagegen sehr wohl. Einen wesentlichen Einfluss haben die Folgen der invasiven Beatmung und das durch COVID-19 verursachte Acute Respiratory Distress Syndrome (ARDS). Hohe Beatmungsdrücke wirken sich negativ auf die Nierenperfusion aus und können so zur Entstehung einer AKI beitragen. Die durch das ARDS verursachte Entzündungsreaktion sowie die für COVID-19 typische endotheliale Dysfunktion in Kombination mit einer Hyperkoagulabilität sind weitere Faktoren, die die Nierenfunktion negativ beeinflussen können.

## Abstract (en)

Coronavirus disease 2019 (COVID-19)-associated acute kidney injury (AKI) is common in critically ill patients. Renal tropism of severe acute respiratory syndrome coronavirus type 2 (SARS-CoV-2) appears to play only a minor role, whereas the pathological inflammatory response associated with severe COVID-19 is highly relevant. Both the consequences of invasive ventilation and acute respiratory distress syndrome (ARDS) caused by COVID-19 have a significant impact on the pathogenesis of AKI. High ventilation pressures compromise renal perfusion and, thus, may contribute to the development of AKI. The inflammatory response caused by ARDS, as well as the endothelial dysfunction typical of COVID-19 in combination with hypercoagulability are further factors that affect the kidney.

## Hintergrund

Seit nunmehr 2 Jahren bestimmen das „severe acute respiratory syndrome coronavirus type 2“ (SARS-CoV-2) und die Coronaviruserkrankung 2019 (COVID-19) weltweit das Geschehen in Krankenhäusern und Intensivstationen. Neben der Lunge scheint auch die Niere vor allem bei schweren COVID-19-Verläufen besonders betroffen zu sein [57].

In dieser Übersichtsarbeit sollen die akute Nierenschädigung (AKI) bei COVID-19, ihre Pathogenese und spezielle Aspekte der Interaktion zwischen Lunge und Niere näher beleuchtet werden.

## Epidemiologie

Die AKI stellt ein häufiges Phänomen in der Klinik dar [38]. Sie betrifft insbesondere kritisch kranke Patient:innen. In der viel zitierten AKI-EPI-Studie aus dem Jahr 2015 trat sie mit einer Inzidenz von 57,3 % bei Intensivpatient:innen auf [26].

Die Berichte über die Häufigkeit von AKI bei einer SARS-CoV-2-Infektion waren zunächst widersprüchlich. Während erste Daten aus China auf eine niedrige Inzidenz hindeuteten [12], folgten einige Studien aus Europa und den USA, die höhere Inzidenzraten von AKI (bis 46 %) aufzeigten mit jedoch teils großer Divergenz [16, 32]. Später wurden auch höhere Zahlen aus China berichtet [27]. Es zeigten sich zudem starke regionale Unterschiede, was unter anderem auf die uneinheitliche Verwendung von Definitionen zurückgeführt werden kann. Eine Metanalyse fand in den USA und Europa eine Inzidenz von etwa 28 % [19]. Deutlich höhere Zahlen wurden bei kritisch kranken COVID-19-Patient:innen berichtet [62]. So fanden Gupta et al. in einer großen multizentrischen Studie eine Inzidenz von 42,8 % für die AKI bei COVID-19-Patient:innen auf der Intensivstation. Eine große internationale Metaanalyse zeigte sehr ähnlichen Zahlen [52]. Eine retrospektive Vergleichsstudie aus den USA kam zu dem Ergebnis, dass bei COVID-19-Patient:innen eine AKI häufiger als bei der Vergleichsgruppe auftrat und auch häufiger bis zur Nierenersatztherapiepflicht fortschritt [17]. Wie bei einer AKI bei Nicht-COVID-19-Patient:innen ist auch die AKI bei einer SARS-CoV-2-Infektion mit einem erhöhten Sterberisiko assoziiert [39, 64]. Als wesentliche Auslöser für das Auftreten von AKI bei COVID-19 wurden zwischenzeitlich mehrere Risikofaktoren wie männliches Geschlecht, Diabetes [33, 35], erhöhter Body-Mass-Index (BMI), arterielle Hypertonie und vor allem eine vorbestehende chronische Nierenschädigung (CKD) identifiziert [23].

## Diagnose der COVID-19-assoziierten AKI

Die derzeit international am häufigsten verwendete Definition zur Diagnose einer AKI stammt aus der Leitlinie Kidney Disease: Improving Global Outcomes (KDIGO) für die AKI aus dem Jahr 2012 und beinhaltet eine Erhöhung des Serumkreatinins oder eine Reduktion der Urinausscheidung [31]. Vor allem bei der frühen Diagnose bei COVID-19 scheinen Urinanalysen wegweisend zu sein. So konnte bei stationären Patient:innen mit COVID-19 und einer akuten Nierenschädigung in 84 % eine Proteinurie und in 81 % eine Hämaturie nachgewiesen werden, bevor die klassischen KDIGO-Kriterien erfüllt wurden [10]. Die Urinveränderungen weisen somit bei COVID-19 sehr früh auf eine akute Nierenbeteiligung hin. Im Sinne eines kürzlich veröffentlichten Konsensusstatements zum neuen Staging der AKI entspricht das einem *AKI-Stadium 1S* (Biomarker positiv, Serumkreatinin und Oligurie negativ) und kann somit auch bei COVID-19-Patient:innen bereits berücksichtigt werden [46]. Der Vorteil von „neuen Biomarkern“ (z. B. NGAL, Zellzyklusarrestproteine, Kim-1) für die Frühdiagnose und Risikoeinschätzung bei COVID-19-assoziierter AKI ist allerdings noch zu definieren.

## Pathogenese

Die Ursachen einer AKI sind bei Intensivpatient:innen meist komplex und oft multifaktoriell. Dabei ist es zielführend, zwischen direkten und indirekten Faktoren zu unterscheiden. Während unter direkten Faktoren die Wirkung der SARS-CoV-2-Infektion auf die Niere selbst zu verstehen ist, beinhalten indirekte Faktoren alle Folgen der durch COVID-19 verursachten Erkrankung und – insbesondere auf der Intensivstation – auch deren Behandlung [57].

Eine besondere Rolle unter den indirekten Mechanismen spielen Interaktionen mit der Lunge, da die invasive Beatmung und das Acute Respiratory Distress Syndrome (ARDS) sowohl unabhängige Risikofaktoren für eine AKI [14] als auch Folge einer schwer verlaufenden COVID-19-Erkrankung sind [29]. Eine direkte Schädigung der Niere durch SARS-CoV‑2 selbst ist Gegenstand zahlreicher intensiv geführter Diskussionen [6, 60].

### Direkte Faktoren – SARS-CoV-2 und die Niere

#### Renaler Tropismus

Inwiefern eine direkte Schädigung der Niere eine Rolle in der Pathogenese der AKI bei COVID-19 spielt, ist noch nicht abschließend geklärt. Das SARS-CoV‑2 bindet über Rezeptoren für das „Angiotensin Converting Enzyme“ (ACE) 2 an die Zelle. Mithilfe der transmembranösen Serinproteinase 2 (TMPRSS2) erfolgt schließlich die Aufnahme in die Zelle mit nachfolgender intrazellulärer Replikation des Virus. Die ACE2-Rezeptoren lassen sich in der Niere in den proximalen Tubuluszellen und den Podozyten nachweisen [47]. In verschiedenen Autopsiestudien wurde Virus-RNA direkt in der Niere nachgewiesen und in Einzelfällen auch im Harn [25]. Ein Virusnachweis korrelierte mit dem Auftreten einer AKI und mit dem Schweregrad der Erkrankung, was auf eine direkte Schädigung hindeutet [9]. Demgegenüber steht, dass bisher noch kein direkter Virusnachweis aus Nierenbiopsien von COVID-19-Patient:innen mit AKI gelang [25]. Die histopathologischen Veränderungen, die in Biopsien gefunden wurden, reichen von Myoglobinausfällungen in den Tubuli, die typisch für Rhabdomyolyse sind, bis hin zur kollabierenden Glomerulosklerose und thrombotischer Mikroangiopathie. Am häufigsten findet sich eine akute Tubulusnekrose, was eher für eine multifaktorielle Ätiologie der AKI bei COVID-19 als für eine direkte virale Schädigung spricht [8, 45, 51].

##### Merke

Auch wenn eine direkte Schädigung der Niere durch SARS-CoV‑2 denkbar ist, weisen die histologischen Untersuchungen von Nierenbiopsien eher auf eine multifaktorielle Ätiologie hin.

#### Hyperkoagulabilität und endotheliale Dysfunktion

Die endotheliale Dysfunktion übernimmt eine entscheidende Rolle in der Pathophysiologie einer COVID-19-Erkrankung, da SARS-CoV‑2 direkt in endotheliale Zellen eindringt und diese schädigen kann [41, 58]. Das Spikeprotein scheint, über eine Herunterregulation von ACE2, eine Schlüsselrolle in der Endothelzellschädigung zu spielen. Diese endotheliale Dysfunktion bzw. Schädigung betrifft neben der Lung auch andere Organe, wie die Niere [59].

Zusätzlich kommt es bei einer SARS-CoV-2-Infektion auch zu einer überschießenden Aktivierung der Gerinnung [7]. Hierbei spielt die Komplementaktivierung eine wichtige Rolle. Komplementspiegel werden durch eine SARS-CoV-2-Infektion beeinflusst [63] und waren bei invasiv beatmeten COVID-19-Patient:innen höher als bei nichtinvasiv Beatmeten [13]. Dies führt zu einem Status der Hyperkoagulabilität und zeigt sich durch erhöhte D-Dimer-Werte im Blut, die auch mit einer erhöhten Mortalität assoziiert sind [65].

Zusammen tragen diese Faktoren (Hyperkoagulabilität und endotheliale Dysfunktion) zur Entstehung von Mikrothromben bei. In Autopsiestudien wurde beispielsweise gezeigt, dass diese Mikro- und Makrothromben bei COVID-19 im Vergleich zur Influenza häufiger sind [36]. Diese Mikrothromben spielen nicht nur bei der Pathophysiologie des ARDS eine Rolle [44], sondern können auch andere Organsysteme, wie die Nieren, betreffen [43]. In der Niere kommen Mikrothromben daher als mögliche Mitverursacher der AKI bei COVID-19 durch die folgenden Perfusionsstörungen in Betracht [36].

##### Merke

Eine COVID-19-assoziierte endotheliale Dysfunktion und Hyperkoagulabilität sind in der Pathogenese sowohl des ARDS als auch der AKI relevante Faktoren.

### Indirekte Faktoren – Folgen der schweren COVID-19-Infektion auf die Niere

Bei einer schweren COVID-19-Infektion kommt es zum ARDS, das oft eine Therapie auf der Intensivstation mit invasiver Beatmung erforderlich macht.

#### Invasive Beatmung und Folgen für die Niere

**Figure 1:**
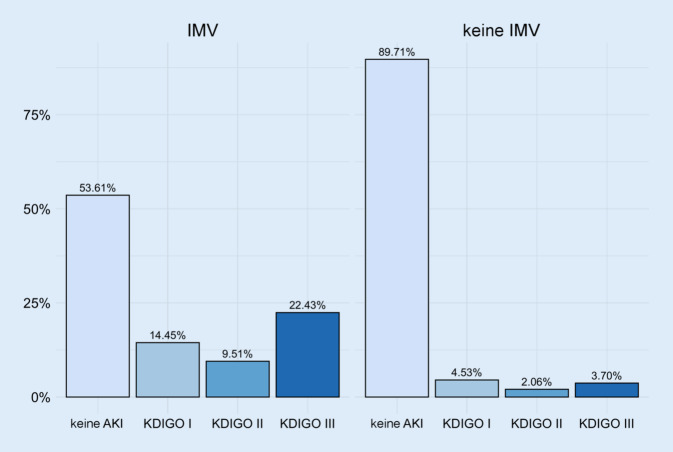

Die invasive maschinelle Beatmung (ohne ARDS) geht mit einer 3‑fachen Erhöhung des Risikos für eine AKI einher [3, 34]. Kohortenstudien zeigten bei COVID-19 ebenfalls einen Zusammenhang zwischen der Rate an invasiver Beatmung und der Häufigkeit von AKI [24, 42]. Einige (retrospektive) Studien lieferten sogar Hinweise, dass der Effekt der mechanischen Beatmung auf die Nierenfunktion ausgeprägter ist als in Nicht-COVID-19-Vergleichsgruppen [18]. Ein Grund für diese Beobachtung könnte die zu Beginn der Pandemie sehr großzügige Indikationsstellung für den Start einer invasiven Beatmung gewesen sein [21, 61]. Dieses Vorgehen, wurde vor allem aufgrund der Angst vor einer raschen klinischen Verschlechterung umgesetzt und dürfte teilweise zu einer längeren Beatmungsdauer geführt haben [32]. Vergleiche der ersten beiden Wellen zeigen zu Beginn (hohe Rate an invasiver Beatmung) auch deutlich höhere AKI-Raten und einen Rückgang der AKI-Inzidenz im Verlauf der Pandemie (Abb. 1, [11, 42]).

Der negative Einfluss der invasiven Beatmung auf die Niere ist Folge hoher Beatmungsdrücke (insbesondere des positiven endexspiratorischen Drucks, PEEP) und daraus resultierender intrathorakaler und -abdomineller Drücke. Durch den erhöhten intrathorakalen Druck wird der venöse Rückfluss zum Herzen behindert, was zu einem verminderten Herzzeitvolumen mit Rechtsherzbelastung und venöser Kongestion führen kann. Verstärkt durch erhöhte intraabdominelle Drücke kommt es so zu einer Verminderung der Nierenperfusion [28]. Darüber hinaus spielen neurohormonelle Effekte eine wichtige Rolle, vor allem vermittelt durch die Aktivierung des Renin-Angiotensin-Aldosteron(RAA)-Systems und des Sympathikus, die vermehrte Freisetzung von antidiuretischem Hormon (ADH) und die Verminderung der atrialen natriuretischen Peptide (ANP/BNP). Alle diese Mechanismen führen zu einem reduzierten renalen Blutfluss, zu einer Reduktion der glomerulären Filtrationsrate (GFR) und schließlich zu einer Oligurie [29].

##### Merke

Eine invasive Beatmung erhöht das Risiko für eine AKI auch bei COVID-19.

#### Acute Respiratory Distress Syndrome

Neben der invasiven Beatmung ist auch das ARDS als Folge einer schweren COVID-19-Erkrankung ein unabhängiger Risikofaktor für die Entwicklung einer AKI. Das Risiko, eine akute Nierenschädigung zu entwickeln, ist bei Patient:innen mit ARDS (Nicht-COVID-19) um ein vielfaches (Odds-Ratio = 11) erhöht [14]. Die Niere reagiert im Vergleich zu anderen Organen besonders empfindlich auf eine verminderte Durchblutung und Sauerstoffmangel. Da das ARDS initial meist zu einer Hypoxämie führt, kann sich dies bereits negativ auf die renale Sauerstoffversorgung auswirken [15]. Zudem lassen sich bei der Beatmung beim ARDS hohe Druckeinstellungen nicht immer vermeiden. Ein hoher PEEP bzw. hohe Beatmungsdrücke können jedoch – wie bereits beschrieben – zu einer schlechteren Durchblutung der Niere führen.

Des Weiteren werden beim ARDS durch das sog. Biotrauma in besonderem Maß proinflammatorische Mediatoren (z. B. Interleukin[IL]-6, Tumornekrosefaktor[TNF]-α und Adhäsionsmoleküle) ausgeschüttet [49]. Deren Freisetzung in den systemischen Kreislauf kann sich negativ auf die Nierenfunktion auswirken. So waren erhöhte Spiegel von z. B. IL‑6, TNF-Rezeptoren und Plasminogenaktivatorinhibitor‑1 unabhängig mit dem Entstehen einer AKI beim ARDS assoziiert [40].

Eine überschießende Immunantwort spielt in der Pathogenese des COVID-19-ARDS eine entscheidende Rolle, wobei zahlreiche inflammatorische Zytokine freigesetzt werden, von denen einige, wie z. B. IL‑6, in direktem Zusammenhang mit einer akuten Nierenschädigung stehen [55]. Obwohl die IL-6-Spiegel bei COVID-19 erhöht sind, ist diese Erhöhung verglichen sowohl mit anderen ARDS-Ursachen [53] als auch mit der bakteriellen Sepsis eher gering [37, 48]. Die Rolle dieser Zytokine im Hinblick auf die Entstehung der akuten Nierenschädigung bei COVID-19 ist daher nicht abschließend geklärt.

##### Merke

Das ARDS und die resultierende systemische Immunantwort wirken sich negativ auf die Nierenfunktion aus.

#### Flüssigkeitsmanagement

**Figure 2:**
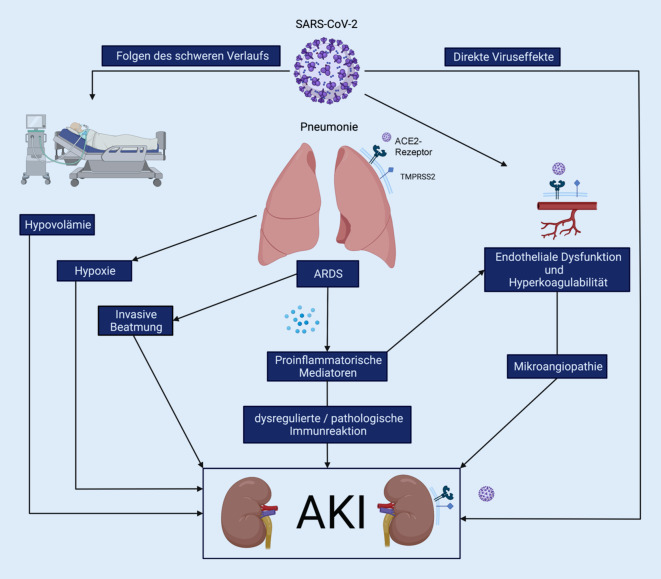

Aufgrund der bereits beschriebenen Mechanismen kommt es unter invasiver mechanischer Beatmung zur einer Salz- und Wasserretention durch die Niere. Zudem wird in dieser Situation zur Behandlung der hämodynamischen Instabilität bedingt durch hohe Beatmungsdrücke und tiefe Sedierung der Pateint:innen mit COVID-19-assoziiertem ARDS vermehrt Flüssigkeit verabreicht. Die Folge ist eine eindeutige Tendenz zur Volumenüberladung. Reduzierte Nierenperfusion und Volumenüberladung begünstigen das Entstehen einer AKI [34]. Restriktives Flüssigkeitsmanagement bei ARDS-Patienten reduziert die Inzidenz von AKI, die Beatmungsdauer und die Mortalität [22] und wurde somit auch in den Surviving Sepsis Guidelines bei COVID-19 empfohlen [4]. Andererseits besteht in der Initialphase einer schweren COVID-19-Infektion oft eine ausgeprägte Hypovolämie. Ein zu rigoroses Befolgen der Flüssigkeitsrestriktion in dieser Phase der Erkrankung könnte diese, für schwere Infektionen typische Hypovolämie noch verstärkt und damit wesentlich zur höheren Inzidenz von AKI in der ersten Welle beigetragen haben [32, 45]. Die derzeitige Strategie beinhaltet eine initial großzügigere Flüssigkeitsgabe bis zur hämodynamischen Stabilisierung, während ein restriktives Regime erst für die spätere Phase des ARDS empfohlen wird (Abb. 2, [4]).

## Therapeutische Aspekte bei COVID-19-bedingter AKI

Derzeit existiert keine spezifische Therapie bei COVID-19-bedingter AKI. Da viele Aspekte der schweren COVID-19-Erkrankung (überschießende Immunantwort, Koagulopathie, Kreislaufversagen) auch in der Pathophysiologie der AKI eine wichtige Rolle spielen, gilt es, diese bestmöglich zu beherrschen.

Dies beinhaltet zum einen eine adäquate Immunmodulation, um eine überschießende Immunreaktion einzudämmen. Der Einsatz von Dexamethason bei sauerstoffpflichtiger COVID-19-Erkrankung gilt mittlerweile als Standardtherapie. So zeigt sich in einer der Arbeiten der Recovery-Studie eine reduzierte Rate an Nierenersatztherapien bei Patient:innen, die mit Dexamethason behandelt wurden (4,4 % vs. 7,5 %; [56]). In einer weiteren Studie der Recovery-Gruppe wurde ähnliches auch für Tocilizumab nachgewiesen [2]. Da die Nierenersatztherapie nicht primärer Endpunkt dieser Studien ist, sind diese Daten nur bedingt aussagekräftig. Dennoch zeigt sich, dass sich eine Immunmodulation bei COVID-19 positiv auf die Nierenfunktion auswirken kann.

Zudem erscheint eine adäquate Antikoagulationsstrategie von wichtiger Bedeutung, um potenzielle Makro- und Mikrothromben (s. zuvor) vorzubeugen. Für die meisten Patient:innen scheint derzeit die prophylaktische Dosierung das Mittel der Wahl zu sein [50], wobei die Empfehlungen zur Dosierung sich während der Pandemie geändert haben. Derzeit gibt es keine ausreichende Datengrundlage, die eine therapeutische Dosierung bei kritisch Kranken rechtfertigen würde [5]. In einer kürzlich publizierten Arbeit der Recovery-Gruppe zur Gabe von Azetylsalizylsäure bei hospitalisierten COVID-19-Patient:innen zeigte sich zwar eine etwas erhöhte Rate an lebend Entlassenen nach 28 Tagen, jedoch kein Unterschied in der Häufigkeit von Nierenfunktionsstörungen [1].

Eine große Bedeutung spielt zudem die Optimierung der renalen Perfusion durch eine adäquate Volumentherapie. Zu Beginn sollte eine Hypovolämie möglichst schnell korrigiert und trotzdem eine weiter bestehende Hypotension frühzeitig mit Vasopressoren behandelt werden. Danach ist ein ausgeglichener Flüssigkeitshaushalt (meist unter Einsatz von Schleifendiuretika) und das Vermeiden einer Volumenüberladung und der damit verbundenen venösen Kongestion anzustreben [30].

Für Patient:innen mit COVID-19-assoziiertem ARDS ist eine lungenprotektive Beatmung, im Sinne eines möglichst niedrigen „driving pressure“ anzustreben. Darüber hinaus sind auch die negativen Effekte des PEEP auf die Hämodynamik und Nierenperfusion zu beachten und somit keinesfalls höhere Werte als die funktionell absolut notwendigen einzusetzen. Die PEEP-Tabelle des ARDS Network kann maximal als Orientierung gelten; in den meisten Fällen können anhand der Compliance bzw. des transpulmonalen Drucks deutlich niedrigere Werte eingestellt werden. [20, 49]. Das gilt vor allem bei zunehmender Beatmungsdauer, bei der es zu einer Zunahme der strukturellen Veränderungen in der Lunge mit Abnahme der Compliance und Zunahme des Totraums kommt [54].

## Fazit für die Praxis

- Ein mit der Coronaviruserkrankung 2019 (COVID-19) assoziiertes Acute Respiratory Distress Syndrome (ARDS) ist mit einer hohen Rate an akuten Nierenschädigungen (AKI) verbunden.
- Bei der Diagnose sollen die üblichen Kriterien (Kidney Disease: Improving Global Outcomes, KDIGO) mit besonderem Fokus auf frühe Veränderungen im Harnstatus angewandt werden.
- Da derzeit keine spezifische Therapie der SARS-CoV-2-assoziierten AKI existiert, ist besonders auf die Optimierung bzw. Vermeidung der verschiedenen potenziellen Auslöser zu achten (Volumenstatus, hohe Beatmungsdrücke, überschießende Inflammation, Nephrotoxine).
- Weitere Studien sind nötig, um direkte virale Effekte (und deren AKI-spezifische Therapie) sowie die Langzeitfolgen einer COVID-19-assoziierten AKI zu untersuchen.
